# Supplementary material for: Are carbon emissions trading and green financial instruments synergistic? -Comprehensive quantitative research based on content analysis
Source: PLoS One. 2024 Mar 7;19(3):e0298601. doi: 10.1371/journal.pone.0298601 (PMC10919601; doi:10.1371/journal.pone.0298601)
Supplement: S1 Data — (ZIP) [file pone.0298601.s001.zip › Data/地级市Z_F工作报告.zip!/╣π╢1⁄2/╢1⁄2▌╕╩╨/└┤╫╘╣1⁄2╓┌║┼í╛┬φ┐╦╩2╛▌═°í┐_2009.docx]

各位代表：

　　现在，我代表市人民政府向大会作政府工作报告，请予审议，并请政协各位委员和其他列席人员提出意见。

2008年工作回顾

　　刚刚过去的2008年，是很不寻常、很不平凡的一年。改革开放30周年、东莞成立地级市20周年的辉煌成就，让我们豪情满怀；北京奥运和神舟七号的成功壮举，令我们心潮澎湃；抗震救灾的举国动员，使我们众志成城。而国内外纷繁复杂的形势，又给我们带来了严峻挑战。一年来，在上级和市委的坚强领导下，我们高举中国特色社会主义伟大旗帜，继续解放思想，深入落实科学发展观，推进经济社会双转型，开拓创新，克难奋进，较好地完成了市第十四届人大三次会议确定的年度目标任务。

　　过去一年，是形势异常严峻、复杂多变的一年。从国际看，能源和原材料价格暴涨暴跌，美国次贷危机引发了全球金融危机，欧美消费市场大幅萎缩，世界经济增长明显放缓。从国内看，严重自然灾害频频发生，人民币升值，劳动力成本攀升，经济增长高位回落，国家宏观政策大幅调整，转向全力保增长。从我市看，东莞正处在矛盾多发期、内部调整期和转型攻坚期，在突如其来的形势变化面前，产业结构不合理、资源主导、外向依赖、抗御风险能力不强的问题更加凸显，经济发展受到严重冲击，尤其第四季度出现了较大回落。这使全市经济社会发展遭遇了新世纪以来最为严峻的考验和挑战。

　　过去一年，是我们迎难而上、沉着应对的一年。面对日益激烈的区域竞争，面对转型发展的艰巨任务，面对金融海啸的巨大冲击，面对危机引发的社会矛盾，我们迎难而上、沉着应战。我们全面总结改革开放的成功经验，坚定了信念，鼓舞了士气。我们继续解放思想，深入实践科学发展观，开展大学习、大讨论、大调研，查摆了问题，明确了任务，落实了整改。我们积极稳妥推进产业结构调整，铺开了各类试点，完善了政策体系，积累了宝贵经验。我们树立世界眼光，确立标杆城市，加强区域合作，积极融入珠三角，着力谋求新发展。我们洞察形势变化，快速决策部署，切实加强对经济工作的领导，全力以赴保增长。我们以就地转型为根本，以财税支持为重点，以改善服务为手段，出台和贯彻了一系列针对性强的政策措施，千方百计帮扶企业渡难关。我们深化专项整治，加强长效管理，有效处置化解矛盾冲突，维护了安定局面，促进了社会和谐。

　　过去一年，是全市稳妥转型、稳中求进的一年。经济发展在逆境中实现较快增长。预计全市生产总值3710亿元，比上年增长14%，来源于东莞的财政收入601亿元，增长11.4%，其中市财政一般预算收入209亿元，增长12.2%。出口总额654.5亿美元，增长8.7%。实际利用外资24.5亿美元，增长15.5%。固定资产总投资942亿元，增长12%。社会消费品零售总额839亿元，增长20.5%。金融机构本外币各项存款余额4460亿元，增长16%。城市居民人均可支配收入30268元，农村居民人均纯收入12328元，分别增长12%和6.2%。居民消费价格总指数上涨6%。产业转型初见成效。第三产业增加值增速快于第二产业18个百分点，三次产业比例从0.4：56.8：42.8调整为0.3：52.6：47.1。一般贸易出口增速快于加工贸易26.7个百分点，民营固定资产投资增速高于外资经济15个百分点。单位GDP耗地下降15%，耗电下降11.6%。社会转型持续深化。综合治理深入开展，管理机制不断完善，社会大局稳定和谐，各项事业全面进步，全国文明城市创建活动成效明显，市民素质不断提高。城市形象有效提升。过去一年，东莞获得了中国制造业名城、国家园林城市、信息产业国家高技术产业基地、省林业生态市等荣誉称号，创建全国社会治安综合治理优秀市工作通过国家检查考评，省双拥模范城实现七连冠，城市环境考核排名从全省第九位上升到第四位，东莞入选中国城市综合创新能力50强，被列为中国改革开放18个典型地区之一。在中国社科院发布的报告中，东莞综合竞争力在全球500座城市中名列第254位。

　　回顾过去一年，我们重点抓了以下八项工作：

　　一、帮助企业扎根发展。切实采取积极措施，推动企业扎根东莞，做大做强。尤其在危机到来之际，加大帮扶力度，稳定企业经营。帮助企业增强信心。深入开展政策宣讲，组织系列帮扶活动，发动市、镇、村三级干部全面走访企业，表明共渡难关的态度，增强企业发展信心，外资企业增资占合同利用外资总额的65.6%，提高6.2个百分点。加强莞港、莞台合作招商，实行对欧登门招商。成立内资经济促进中心，强化内资引进。帮助企业减轻资金压力。设立10亿元融资专项资金，以财政贴息和风险补偿相结合的方式，帮助企业解决融资难问题。取消或降低多项收费，共为企业减负约10亿元。推动口岸保税物流中心建设，降低企业物流成本。帮助企业缓解资源瓶颈。积极向省争取网电指标；市财政借款2.5亿元、补贴1093万元，支持地方电厂发电，缓解用电供需矛盾。统筹年度新增用地指标1.2万亩，保障优质项目和重点民生工程用地。帮助企业拓展国内市场。在100家外资企业开展内销试点，建立内销“快速通道”，全市外资企业内销总额1673.6亿元，增长19.5%，一定程度上缓解了出口订单减少的影响。

　　二、推进产业结构调整。把结构调整升级作为落实科学发展观最核心的任务，积极稳妥推进。以各类试点为突破。制订《关于推进产业结构调整促进产业转型升级的意见》等“1+26”政策文件，铺开了镇、村、行业、企业等4大类共62个试点的工作，有关试点在“退二进三”、技术改造、创建名牌等方面初见成效。认定上市后备企业15家，新增上市公司1家。以加工贸易转型升级为重点。设立10亿元专项资金，加强政策指导服务，帮助加工贸易企业就地转型升级。全市来料加工企业转“三资”企业和民营企业197家，外资企业设立研发机构12个，内销品牌215个。东莞被列为省加工贸易转型升级试点城市。以园区建设为龙头。松山湖全年引进项目105个，合同引资85.4亿元。虎门港5号、6号泊位建成投产，获批的深水泊位增至17个，成为对台直航首批港口之一，保税物流中心（B型）获国家批准设立。东莞生态园12项基础工程动工建设。长安滨海新区规划建设前期工作开始起步。以节能减排为硬约束。拒批污染项目371个，关闭“四纯两小”企业149家，加强企业排污监控，深化养殖业污染等整治。组建能源利用检测中心，落实重点耗能企业节能任务。修编土地总体规划，出台节约集约利用土地实施细则。实行阶梯水价，促进节约用水。以产业和劳动力双转移为推动。与惠州、韶关合作筹建两个市级产业转移园，接收我省东西北地区5100名学生到我市就读职校，接收劳动力就业33.6万人。

　　三、增强自主发展能力。扎实推进“科技东莞”、“创业东莞”工程，增强企业竞争力和内源经济实力。强化政策引导。落实和完善科技政策，筹资3亿元组建市科技创业投资公司，首设“百万元市长奖”，发放1.46亿元免息小额贷款，扶持创新创业，激励发明创造。全市专利申请量和授权量分别居全省第二、第三位，其中发明专利授权量增长1.5倍，首获国家专利金奖1项。加快平台建设。建成公共技术创新平台6个。新增省级企业工程中心和技术中心11个，总数达34个。新认定省专业镇和专业镇技术创新平台各2个，总数分别达11个和10个。新增企业博士后科研工作站4家。培育“两自”企业。获批国家高新技术企业153家。新增省民营科技企业55家，总数470家。获国家和省资助项目110个。全市高新技术产业工业总产值2403亿元，占全市工业总产值的33.4%。深入实施名牌带动战略，新增省级以上名牌名标65个，累计376个，参与制定修订国家标准和行业标准68个。新增各类人才1.7万人。壮大民营经济。落实“新48条”，建立民营企业排忧解难协调制度，推动民营经济发展，全市民营单位注册总户数48万户，注册资金925亿元，分别增长6%和12%。

　　四、强化公共安全管理。坚持专项治理与加强防控相结合，积极维护社会稳定。着力加强社会治安。深化“三基工程”建设，推进警力下沉，基层一线警力占总警力89.1%。完成110、119、122“三台合一”首期工程，在用各类视频监控点12.8万个，东莞获评全国科技强警示范城市。加强群防群治，严打违法犯罪，深化“治摩禁电”，治安形势保持平稳，飞车抢夺案下降42.2%，涉摩交通事故下降27.3%。着力整治安全隐患。开展隐患治理年行动，继续狠抓安全生产，深入推进“三小”场所、出租屋、非法行医、无证照经营、危旧桥梁厂房、产品质量和食品药品安全等专项整治，全市火灾事故宗数、死亡人数分别下降22.7%和75.6%。着力化解矛盾纠纷。扎实开展重信重访专项治理和市镇领导大接访活动，大力加强劳资调解，有效化解了一批群众关注的热点难点问题，群众越级到省上访批次下降9.5%，劳动争议案件调解率80.6%。

　　五、加大城乡统筹力度。坚持以工促农、以城带乡，促进区域协调发展。加快基础设施建设。26项市属道路交通和城市功能配套重点工程顺利竣工，30项开工建设。完成电网建设投资18.8亿元，新增LNG管网105公里，建成水利防灾减灾工程164项。积极扶持镇村发展。市财政共安排42.9亿元支持镇村各项建设，减轻村组负担。32个镇街可支配收入206亿元，增长30.3%。全市村组两级可支配收入181亿元，增长3.5%；净资产852亿元，增长6%。对573个村进行经济责任审计整改。完成村（居）委会换届选举。努力改善城乡环境。划定1103平方公里的生态控制线。建立生态补偿机制。完成生态环境整治工程146项，新增绿地699万平方米。在336个村（社区）铺开了旧村整治。市财政补贴1.54亿元建设农村公厕和垃圾转运站。运河综合整治稳步推进。开展违法用地专项整治，拆除违章建筑面积108万平方米，复耕复绿1.6万亩。

　　六、全面发展社会事业。坚持经济社会同步建设，大力推动各项社会事业发展。促进教育优质均衡发展。“三转二”办学体制改革稳步推进，东莞职业技术学院一期竣工，各级各类教育质量全面提升。全市普通高考录取率84.3%，每万户籍人口升大学92人，在全省地级以上市中名列第一。促进文化事业繁荣发展。创建全国文明城市通过国检。“文化三城”建设和公益性文化事业单位改革稳步推进，岭南画院、可园博物馆、文学艺术院正式启用，举办了首届“岭南美术节”，承办了广东省第四届音乐舞蹈花会、国际花园城市决赛等活动。促进社保、体育、卫生、人口计生等事业协调发展。继续抓好社保扩面征缴，全市各险种参保总人次1951万，增加503.6万。最低工资标准从690元提高到770元。推动全民健身运动，全市新增体育场地面积264万平方米。基本完成市人民医院新院、妇幼保健院新院、第三人民医院、疾病预防控制中心的主体工程建设。改革社区卫生服务管理体制，实现向“政府办政府管”的转变。加强人口和计生管理，人口自然增长率6.2‰。援助广西河池、西藏林芝、新疆哈密、重庆巫山及韶关新丰、乳源等对口帮扶地区财物9051万元。国防人防、气象测报、科普法普、外事侨务、工青妇幼、民族宗教、统计审计、档案方志以及打私、老龄等工作扎实推进。

　　七、坚持办好民生实事。以办好十件实事为重点，推进民生工程建设。一是创建平安社区。新建社区警务室127个，实现社区全覆盖。建成平安社区100个。二是优化公交服务。投入1.46亿元，新建一批公交站场、站亭和站台，增加了公交班次，延长了服务时间。三是加强住房保障。筹集建设廉租房17573套、经济适用房3107套；规划安排18亿元改造渔民社区。四是推动村际联网路改造。完成33条，总长115公里。五是加快治污工程建设。基本建成污水处理厂16座、截污主干管网工程13项。六是完善社区卫生服务。318个公办社区卫生服务机构建成并投入使用。七是健全基本医疗保障。为住院基本医疗保险参保人增加了普通门诊医疗保障。八是落实教育减负。对困难家庭高中生和大学生提供补助，低保家庭学生分别提高到3000元和7000元；为全市中职一、二年级户籍和部分非户籍学生提供1500元助学金。免收义务教育阶段户籍借读生杂费差额。九是帮扶困难群众。最低生活保障标准提高到400元。向19.9万名户籍困难群众发放每人1000元临时生活补贴。向1.9万名残疾人提供专项补助金以及康复、就业服务。市财政安排1.18亿元用于全市低温洪涝灾害救灾复产。十是促进培训就业。市财政补贴5859万元，资助技能培训11.7万人次。

　　八、着力提高行政效能。以提高行政效率为主线，强化执行能力，优化政府服务。积极创新行政管理。推进第三轮行政审批制度改革，调整行政审批事项108项，取消10项。探索并联审批，下放审批权限。46个单位和17个镇街设立了“一站式”办事窗口。成立新莞人服务管理局，城市综合执法管理局挂牌运作。健全监督考核体系。制定镇街领导班子和领导干部落实科学发展观评价考核办法。从预算编制、审批、执行等环节入手，加强财政绩效评价管理。完成行政审批电子监察系统三期工程，在全省率先实行审批绩效测评结果向社会公布。机关效能投诉减少22.6%。加强机关作风建设。严格执行党风廉政建设责任制，完善各项制度，整治行业风气，继续开展“市民评机关”活动。深入调查研究，形成了一批调研成果，有效增强了政府工作的系统性和主动性。

　　此外，我市支援汶川地震灾区抗震救灾和恢复重建工作也取得了显著成效，派出救援支援队伍200多人次，收治伤病人员21名，接收学生就读359名，全市累计捐款捐物7亿多元，援建板房18104套。对口支援映秀镇恢复重建第一批6个项目已经启动。

　　各位代表！回顾过去一年，我们倍感成绩来之不易。在严峻复杂的形势下，我们迎难而上，奋力攻坚，在转型发展的征程上迈出了坚实的步伐。这些成绩的取得，离不开上级和市委的正确领导，离不开市人大、市政协的监督支持，离不开广大投资者的勤奋经营，离不开全体市民和驻莞部队的共同努力，离不开港澳台同胞、海外侨胞以及国际友人的关心帮助。在此，我们表示衷心的感谢！

　　回顾过去一年，我们也清醒地认识到，全市经济社会发展还存在不少问题和隐忧。经济下行的压力加大，出口增长放缓，利用外资减速，固定资产投资回落；企业经营较为困难，生产成本上升，订单有所减少，关停企业数量有所增加；农村集体增收难度大，收不抵支矛盾显现；资源环境问题仍然突出，社会管理任务十分繁重，等等。充分暴露了发展方式仍然粗放、产业结构不合理、自主创新能力不强、体制机制不够完善等深层次问题，迫切要求我们重视解决。

　　回顾过去一年，我们还得到了许多有益的启示。我们深深地体会到：要应对危机和战胜困难，必须坚定科学发展、转型发展的决心。实践证明，转变经济发展方式是提高抗御风险能力和核心竞争能力的根本途径，要毫不动摇地推进结构调整，推进自主创新。必须坚定以世界眼光谋划发展的理念。今日之东莞，是中国东莞，也是世界东莞，没有开阔的视野和创新的思维，就无法找准定位，决胜未来。必须坚定以制造产业立市的方向。美国次贷危机表明，实体经济是持续发展的重要支撑。制造业是东莞赖以生存和发展的根基，要坚定不移地建设以信息产业为特色的现代制造业名城。必须增强防范经济风险的意识。开放型经济的风险突发性强，波及面广，我们要切实强化风险意识，完善防范机制，确保经济安全。

总体形势与工作要求

　　当前，国际金融危机的影响仍未见底。只有科学研判形势，明确工作要求，积极有效应对，才能趋利避害，在新一轮的发展中赢得主动。

　　首先，我们仍然面临严峻的困难挑战，必须清醒认识。应该看到，国际金融危机已经从局部发展到全球，从金融领域扩散到实体经济领域，世界经济增长明显减速。随着外部需求的萎缩和跨国企业扩张步伐的放缓，我市外贸出口将继续受到影响，招商引资工作的难度也将加大，经济增长的外部拉动作用明显不足。应该看到，去年受冲击的主要是直接出口企业，接下来将波及更多上下游配套企业，各级财政特别是村组集体经济增收的困难不小。因经济减速、企业减产或关停，也容易引发劳资纠纷、失业等系列社会问题。应该看到，金融危机造成消费者信心不足和消费预期下降，股市、房市、车市相继受挫，市场消费短期内难以有大的增长。2008年我市经受的冲击主要集中在第四季度，但2009年我们将要面临的，很可能是全年的困难。我们必须把情况掌握得更全面一些，把困难估计得更充分一些，做好艰苦奋战、应对挑战的准备。

　　其次，我们拥有克服困难的有利条件，必须坚定信心。面对困难，信心至关重要。我们要坚信，虽然国际经济金融发展遇到了困难和挫折，但世界和平发展的主题、经济全球化和技术革命的趋势没有改变。世界各国纷纷出手救市，对稳定经济形势、增强消费信心将起到积极的作用。我们要坚信，我国综合国力日益增强，集中力量战胜困难的优势明显，当前经济基本面良好，金融体系稳健，政治社会稳定，国内市场广阔，劳动力资源丰富，在国际竞争中具有较大优势，国际社会仍然看好中国。特别是中央迅速出台了一系列刺激经济政策，4万亿元的项目投资，必将有效拉动经济增长。我们要坚信，经过改革开放30年的发展，东莞拥有了比较雄厚的财力物力，构筑了相对完善的市场机制，积累了发展经济的丰富经验，锤炼了奋发有为的优秀干部和企业家队伍，为应对危机打下了坚实的基础。我市下来1400亿元的投资拉动，将在保增长上发挥重要作用。当前东莞的地缘人缘商缘优势仍在，产业配套优势仍在，企业生产能力仍在，市场基本需求仍在。而诸多不利因素正在化解，消费物价涨幅回落，资源瓶颈有所缓解，尤其是加工贸易出口退税率的提高、增值税的转型、银行利率的下调，燃油、钢材等原材料价格的大幅下降，将有效缓解企业经营压力。只要我们充分发挥优势，积极妥善应对，就一定能够战胜困难。

　　第三，我们处于不进则退的关键时期，必须主动作为。危机当前，不进则退。我们必须以非常的工作状态和精神状态，认真落实中央、省、市应对困难的各项措施，确保经济平稳较快增长。我们必须善于在危机中抓住机遇，在逆境中推动发展。尤其要认识到，当前的危机，是危中有机，是传统发展模式之危，科学发展模式之机。实践证明，资源主导、外向依赖的发展模式，不仅直接带来了资源环境代价沉重、社会管理压力巨大等问题，而且抗御风险能力不强，无法承受国际市场波动、国内政策调整造成的冲击。实践证明，凡是转型升级早的企业，市场竞争能力就强，在危机面前就显得主动。在危机的倒逼下，广大干部群众进一步认识到科学发展的重要性，进一步增强了转型发展的主动性。广大企业切实感受到了转型升级的必要性，切实强化了改变经营策略、提高自主发展能力的积极性。这种转变就是转机，是统一思想、坚定信念之机，是加快转型、增创优势之机。我们必须因势利导，更加积极地推进结构调整，推动转型发展。

　　总之，办法总比困难多。我们必须积极主动，出手要快，出拳要重，措施要准，工作要实。今年政府工作的总体要求是：高举中国特色社会主义伟大旗帜，全面贯彻中央、省的重要会议以及市委十二届四次全会精神，深入实践科学发展观，推进经济社会双转型。坚持应对危机与化危为机相结合，切实落实“三促进一保持”要求，把保持经济平稳较快增长作为首要任务，把结构调整升级作为根本出路，把加强经济社会管理作为重要保障，把改善民生作为出发点和落脚点，着力保增长，积极促转型，全面抓管理，更加重民生，确保全市经济社会又好又快发展。

　　具体来讲，保增长，就是要实施积极的财税金融政策，通过加大基建投资、稳定企业经营等多种手段，最大限度地扩大内需、刺激消费、稳定出口，保持经济平稳较快发展。促转型，就是要积极促进自主创新、促进传统产业转型升级、促进现代产业体系建设，确保转型发展的方向不变、力度不减、步子不停。抓管理，就是要加强宏观经济形势研判，密切监控经济运行状况，强化财政收支和集体资产管理，切实防范和应对经济风险；加强隐患排查整改，及时化解经济减速时期可能增加的矛盾纠纷，维护社会稳定和谐。重民生，就是要着力发展普惠型、共享型的社会事业和保障体系，认真解决社会热点难点问题，减轻危机对群众生活的影响。

　　综合考虑各方面因素，今年全市经济社会发展的目标是：生产总值增长10%；市财政一般预算收入增长8%；固定资产投资总额增长16%；社会消费品零售总额增长14%；力争外贸出口有所增长；居民消费价格总指数上升控制在4%以内；城镇登记失业率控制在3%以内；城市居民人均可支配收入增长6%；农民人均纯收入增长5%；单位生产总值能耗下降4.4%；人口自然增长率控制在6.4‰以内。

2009年工作安排

　　为了实现年度目标任务，具体要抓好以下九个方面工作：

　　一、着力加强扶企引商，多策并举共度时艰

　　动员全社会力量，团结携手，共度时艰，突出稳定企业经营，促进经济平稳增长。

　　营造帮扶企业氛围，千方百计稳定企业信心。密切关注宏观经济运行趋势，及时主动决策应对。密切关注企业经营，及时介入帮扶指导。继续举办“稳定企业扎根发展”系列活动，完善落实各项企业服务制度，充分发挥商会和行业协会沟通政企、服务企业的作用。加强政策宣传，树立正面典型，推广成功经验，营造政府帮扶、企业自强、社会支持的良好氛围。

　　狠抓扶持政策落实，想方设法帮助企业解困。认真落实中央、省、市各项扶持政策，突出抓好我市“五个10亿”计划的细化实施，积极帮扶中小企业，扩大政策普惠面。加快解决产权抵押问题，有效盘活沉淀资产，增强金融机构放贷积极性，用好四大银行2100亿元的授信贷款。鼓励企业拓展市场，实行政府采购向在莞企业倾斜。坚决清理不合理收费，有效减轻企业负担。

　　更加注重招商引资，内外并重引进优质项目。细化招商目录，针对大型项目、产业链缺失项目、产业服务项目，主动组织各种招商活动，突出加强对港澳台、日韩、欧美招商，奖励引资贡献突出的单位和个人。抓住我市被确定为落实CEPA及先行先试政策措施试点市的机遇，大力引进港澳现代服务业。加大内资引进力度，加强与周边地区的产业合作。

　　二、稳妥调整产业结构，构建现代产业体系

　　贯彻落实《珠三角地区改革发展规划纲要》，积极推进产业结构调整升级，主动融入珠三角区域经济一体化发展。

　　坚定不移地谋求重点领域突破。总结推广试点经验，出台实施“1+26”政策文件，整合优化各项扶持措施，向重点行业企业倾斜，着力扶优扶强。突出扶持电子信息等先进制造产业，加快松山湖IT研发园建设，积极发展平板显示、新光源、新能源和新型移动通信等产业。突出扶持现代服务产业，加快发展金融、物流、会展等产业，稳步发展服务外包、创意产业和总部经济。突出扶持重点企业，各镇街分别从优势产业中筛选5—8家企业，进行重点扶持，促进做大做强。突出打造镇村转型典范，对原5个试点镇继续落实“三优先”，力争在优质项目引进、“退二进三”、集体经济转型等方面取得新的突破。

　　坚定不移地促进加工贸易转型。继续推动来料加工企业不停产转“三资”企业，鼓励资助拓展内销、技术改造、设立研发机构和地区总部。支持港台产业服务机构进入东莞，直接为企业转型升级提供服务。协调解决企业内销碰到的突出问题，探索建立外资企业产品直销中心，继续做好内销试点工作。加快“两仓合一”保税物流发展，推动加工贸易合同电子审批全覆盖。

　　坚定不移地推动产业集群升级。积极改造提升传统优势产业，继续抓好长安五金模具、虎门服装、大朗毛织、大岭山家具、石龙电子等省级产业集群升级示范区建设，推动特色产业、专业市场、会展经济互动发展。加快专业镇技术创新平台建设，大力创建区域品牌。

　　坚定不移地落实节能降耗减排。深入创建国家环保模范城市，控制污染项目引进，加强企业排污监管，开展污染专项整治。抓紧9个环保专业基地的规划、报批和建设。加强项目节能评估审查，深化企业节能行动，大力推广节能路灯。积极开展省循环经济和国家节水型社会建设试点工作。推广应用天然气、水煤浆等清洁能源，新建一批汽车供气站，加大清洁生产执法力度。深入推进依法集约节约用地，落实土地利用总体规划，加强土地执法监察，严守耕地保护红线，继续清理盘活闲置用地。

　　三、积极推进自主创新，增强核心竞争能力

　　加大引导扶持力度，弘扬艰苦创业精神，营造全民创业氛围，激发企业创新活力，推动自主创新发展。

　　大力培育“两自”企业。落实企业研发费税前抵扣等优惠政策，用好我市多个财政专项资金，对认定的高新技术企业、民营科技企业，给予重点扶持。鼓励企业设立研发机构，增加研发投入，加快技术改造，开发专利产品。鼓励企业争取重大科技专项，对国家级、省级资助项目给予配套支持。落实省“千百亿名牌培育工程”，扶持企业争创各类名牌名标。积极参与行业标准、国家标准的修订制定。

　　大力推动产学研合作。加强科研院所与行业、企业的有效对接，加快东莞中山大学研究院、工业和信息化部第五研究所东莞分中心等建设。组建发展省部产学研创新联盟。认真实施省部企业科技特派员行动计划。大力推进节能减排与再生资源、产业共性技术等省重大科技专项，积极配合中国散裂中子源项目。办好东莞国际合作周等科技交流活动。

　　大力优化创新创业环境。积极开展省科技金融试点工作，促进科技与金融结合，发展创业投资，推进企业上市。加快产品质量检测基地等各类公共平台和行业技术平台的建设。加大知识产权保护力度。推进集体福利分配制度改革，落实小额低息免息贷款、税费减免等政策，鼓励就业创业。放宽企业人才入户标准，简化落户审批和办理程序，在各镇街和市级园区成立新型社区居委会，解决基层入户难问题；帮助人才解决配偶就业、子女入学等实际困难。继续资助技能培训，提升劳动者素质。

　　四、加大园区开发力度，培育经济新增长点

　　加快园区载体建设，迅速形成产业规模，强化辐射带动作用，使园区成为全市发展的重要引擎。

　　加速松山湖的产业集聚。把壮大产业实力作为当前最突出的任务，真正发挥全市科学发展示范区、产业升级引领区的作用。以市级审批权限下放为契机，全力加快引资步伐，强化跟踪服务，推动已签在建项目早日投产，加快集聚一批高新技术企业和产业支援服务企业。继续推进科技园二期、创意生活城、生态景观带等建设，积极做好国家级园区申报工作。

　　推动虎门港的全面提速。积极支持港区主码头泊位的建设运营，全面推进保税物流中心建设，积极申报综合保税区，提升港口核心功能。切实加强征地拆迁工作，推动立沙岛整体开发。成立对台直航促进机构，加速莞台航运合作。推进虎门港资本运营，优化港口开发模式。

　　加快东莞生态园和长安滨海新区的基础建设。加快完善东莞生态园规划体系，完成收地清拆任务，抓紧已动工的12项工程，全面启动拟开工的19项工程，基本完成主要路网建设。加快长安滨海新区的规划编制和申报，尽快启动水利、道路、填海等项目建设。

　　推进镇村工业集聚区的整合提升和产业转移园区建设。研究统筹东部工业园发展，实行统一规划管理，提升开发建设水平。加强镇村工业集聚区的整合改造，优化厂区布局，完善功能配套。推动与韶关、惠州共建市级产业转移园，争创全省示范性产业转移园区。

　　五、加快基础设施建设，有效扩大投资拉动

　　把基础设施建设作为拉动投资、扩大内需的重要抓手，全力加快在建筹建项目进度。

　　加快推进道路交通项目建设。加快建设轨道交通，力争年内动工东莞轻轨首期工程，配合穗莞深城际轨道干线东莞段建设。加快完善高快速路网，建成环城路与广深高速石鼓连接线、环莞快速路首期、莞深高速三期、常虎高速新联支线、惠常高速东莞段，推进深圳外环高速东莞段、博深高速、从莞高速、常虎高速虎门港支线二期、东莞大道延长线等建设。加快石大公路、中麻公路、中望公路、谢樟公路等市域公路建设改造。加快镇村联网路建设。

　　加快推进城市功能配套项目建设。加强电网建设改造，建成110千伏及以上输变电工程16项，新增主变容量344万千伏安。推进供水管网改造，完成第六水厂优质供水工程，加快东江与水库联网工程。新建LNG管网160公里。加快水利防灾减灾工程建设，完成前两批共365宗，启动第三批70宗。建成市人民医院新院、妇幼保健院新院、第三人民医院、疾病预防控制中心、广电中心等项目，动工市中医院新院、卫校新校、东莞篮球中心等项目。

　　加快推进生态环境工程项目建设。基本完成污水处理工程，启动支次管网工程，建成医疗废物处理中心和污泥处理厂，加快5座生活垃圾处理厂的建设。按照“截污、清淤、活源、治堤”八字方针，扎实推进运河综合整治。深化农村环境“五整治”，提高生态环境质量。

　　六、加强基层统筹管理，加力扶持镇村发展

　　坚持“统筹、放权、扶持”有机结合，积极推进体制机制改革，促进镇村持续稳定协调发展。

　　以“三旧”改造为突破，有效整合发展空间。以省“三旧”改造试点为契机，出台实施暂行管理办法，对转型企业土地权属、建设用地改变用途、成片拆迁改造等进行分类处置，着力解决历史遗留问题，简化操作程序，调动各方参与的积极性。加大统筹推进力度，结合产业结构调整，制订镇一级“三旧”改造规划实施方案，倡导农民公寓多村联建，推进工业进园，鼓励“退二进三”。

　　以转移支付为激励，增强镇村发展动力。完善镇街“超收奖励”的财政激励机制。积极探索村级体制改革，逐步分离村级行政管理与经济运营职能。继续实施专项扶贫政策，重新划定经济欠发达镇、村，对新列入的欠发达镇提供连续3年每年1亿元扶贫贴息贷款；对新列入的欠发达村提供300—700万元扶贫贴息贷款, 贴息3年，一次性增加50—100亩的用地指标；对原11个欠发达镇的扶贫贷款统一延期贴息3年。市财政安排2.3亿元，对经济实力排名靠后的285个村（社区）公共管理支出进行补助。

　　以资产管理为重点，促进增收节支减债。严格执行重大事项审查、土地款管理、经济合同管理等制度，强化镇街内部审计和农村审计监督，切实加强镇街财政收支和农村集体资产管理。严控一般性支出和楼堂馆所建设，严控举债推进基础设施建设，严控分红过快增长，严控低价出让集体资产，规范村一级担保贷款，积极防范集体债务风险。大力完善集体经济组织公司化管理，实现资产保值增值。及时做好关闭企业厂房设备的盘活利用。

　　以生态高效为方向，加快现代农业发展。积极推动土地承包经营权流转，加强农业机械化推广，促进适度规模经营。力争全市农业龙头企业达20家以上，新型农民专业合作组织达25家以上。基本完成10个市级农业园区的基础设施建设，建成现代标准农田7万亩，建设市级特色农业标准化示范基地4个。完善农业补贴机制，开展农产品产销联建，加强主要农产品的应急储备。开展农业政策性保险试点。加快渔民社区的规划改造。

　　七、强化城市综合治理，维护社会安全稳定

　　把维护稳定作为第一责任，加大治理力度，提高管理水平，营造安定有序的社会环境。

　　连续作战，推动治安形势持续好转。坚持夯实基层，继续推进警力下沉，加大路面巡逻力度，加强出租屋和流动人口管理，新建平安社区200个。坚持严打高压，重拳打击恶性犯罪和多发性犯罪，严厉打击老虎机、地下“六合彩”等赌博行为以及娱乐场所涉毒涉黄行为。坚持科技强警，推进公安系统信息化建设，继续完善视频监控网络。坚持从优待警与从严治警相结合，提高队伍综合素质。

　　毫不松懈，全面加强安全生产监管。执行最严格的安全生产和食品药品质量安全标准，继续深化专项整治，突出抓好危险化学品、人员密集场所、“五小”经营单位等重点领域，严厉打击非法生产经营，切实消除安全隐患，坚决遏制重特大事故发生。建立重大危险源监控预警系统，推动化工重点规划区域、安全农产品加工配送中心、镇街食品生产基地等建设。夯实基层消防队伍，在全市所有村（社区）建立安全办。加强安全宣传教育培训，探索安全生产管理市场化运作模式。

　　疏堵结合，继续优化市政交通管理。加强城市管理综合执法，大力整治城市“六乱”。深入推进“治摩”工作，禁止摩托车在环城路以内的核心城区行驶。坚持公交优先发展，完善市区、镇内和跨镇三级公交网络，抓紧快速公交试验段和公交站点建设，增加公交运力投放。加强出租车行业的扶持和管理。加快无障碍自行车道建设，完善人行过街设施，缓解市民出行难压力。

　　协调联动，及时排查化解矛盾纠纷。加强和改进信访工作，着力抓好重信重访专项治理，及时有效处理群众反映的问题。深入开展普法教育，加强司法援助和司法调解，引导群众依法维权。加大劳动监察力度，完善企业倒闭风险预警机制，探索建立应急补偿金机制，有效处置因企业问题引发的群体性事件，严厉打击骗贷逃匿、欠薪欠租逃匿等违法行为。加快应急平台一期和市镇两级应急救援指挥中心建设，健全预警体系和应急处理机制，不断提高处置突发事件的能力。

　　八、全面发展社会事业，推动民生持续改善

　　积极发展以民生为重点的各项社会事业，扎实推进民心工程，不断改善群众生活，促进社会文明。

　　坚持优化发展教育事业。深化“三转二”办学体制改革，加快高中阶段学校布局调整，抓紧新建扩建12所高中学校。大力发展职业教育，加快生态园职教城的建设，支持重点职校与我省东西北部市县开展联合办学。完善促进民办教育发展的财政政策。构建教师专业成长体系，培育优化师资力量，年内分类解决代课教师问题。研究制定新莞人子女接受义务教育的管理办法。继续支持高等教育发展。

　　坚持繁荣发展文体事业。巩固全国文明城市创建成果，深化文化事业单位改革，推进“文化三城”建设，大力发展文化产业。继续抓好公益文化活动项目招商。加快工人文化宫和市民艺术中心建设。加强文物和非物质文化遗产保护，充分挖掘虎门鸦片战争遗址、篮球城市等独特资源，打造富有东莞特色的文化旅游品牌。支持文艺创作活动。加强文化市场综合执法。实施“半小时体育生活圈”工程，完善各项体育设施，推动全民健身运动。认真备战第十三届省运会。

　　坚持全面发展卫生、社保、民政等各项事业。深化卫生体制改革，加强社区卫生服务机构管理，加快完善社区卫生服务六大功能。深入推进农（居）民养老保险制度改革，探索建立养老基金支付风险预警机制。调整提高医保年度最高支付限额，住院险从4万元提高到10万元，综合险从8万元提高到15万元，增加生育医疗待遇。继续推进“六好”平安和谐社区建设。引导社会组织发展，建立社会工作制度。坚决完成对口支援汶川映秀镇恢复重建年度任务，争创恢复重建样板工程。认真做好国防人防、双拥共建、人口计生、科普法普、区域协作、外事侨务、港澳台务、工青妇幼、老龄残疾、民族宗教、打私、气象、统计、档案、方志等工作。

　　坚持为群众办实事、办好事。做好大中专毕业生和失业人员的就业服务，落实资助性就业培训、公益性岗位援助等措施。落实最低生活保障和社会救助等制度。继续推进廉租房和经济适用房建设。加快市区和各镇中心区内涝整治。加强生活饮用水卫生监管，基本完成欠发达村老化水管改造。推行户籍人口免费孕检。年内建成32个样板农贸市场。深入开展关爱新莞人活动。今年继续为群众办好十件实事。

　　九、深化政府自身建设，打造务实高效政府

　　创新管理方式，改进机关作风，狠抓工作落实，为应对危机、转型发展提供坚强保障。

　　改革创新促提效。深化行政审批制度改革，加大审批权限向基层部门和镇街下放的力度。对财政投资项目“一书两证”等实行并联审批，确保重点项目优先办理。完善财政投资项目代建制。对重点企业试行筹建期核发临时营业执照制度。加强财政资金使用绩效评价，试行市级财政专项资金竞争性分配改革，切实提高专项资金的使用效益。

　　改进作风优服务。推进电子政务建设，严格落实一次性告知、首问责任等制度，积极推广办事“一条龙服务”。探索建立“一卡通”集中缴费智能系统。整合投诉平台，畅通投诉渠道，集中排查解决一些社会反映强烈的机关作风问题，坚决纠正行业不正之风。严格控制会议、文件数量，严格控制由财政出资举办的晚会、展览、庆典、论坛活动，腾出更多的精力和资金来扶持企业、改善民生。坚持深入基层，推广现场办公，解决实际问题。继续开展公务员行为规范和职业道德教育实践活动，建设为民、务实、清廉的干部队伍。

　　严格督查保落实。强化政府工作的分解执行，明确目标进度，创新方法举措，严格督查督办。落实重大产业项目和基建项目市镇领导挂钩督导制，建立重大问题报告制度和协调处置联席会议制度。实施新修订的工作考评办法，突出对年度重点任务的考核。加强效能监察，落实责任追究，深入推进廉政建设和反腐败斗争。继续开展“市民评机关”活动，主动接受人大、政协和社会的监督。

　　各位代表！在当前这样一个特殊的时期，我们更加需要坚定信心促发展，凝心聚力谋转型。我们坚信，有上级和市委的坚强领导，有人大政协的监督支持，有全市人民的共同努力，东莞一定能够克难奋进，续写新的辉煌！
